# Early Prediction of Left Ventricular Reverse Remodeling in First-Diagnosed Idiopathic Dilated Cardiomyopathy: A Comparison of Linear Model, Random Forest, and Extreme Gradient Boosting

**DOI:** 10.3389/fcvm.2021.684004

**Published:** 2021-08-04

**Authors:** Xiangkun Xie, Mingwei Yang, Shan Xie, Xiaoying Wu, Yuan Jiang, Zhaoyu Liu, Huiying Zhao, Yangxin Chen, Yuling Zhang, Jingfeng Wang

**Affiliations:** ^1^Cardiovascular Medicine Department, Sun Yat-sen Memorial Hospital, Sun Yat-sen University, Guangzhou, China; ^2^Guangdong Province Key Laboratory of Arrhythmia and Electrophysiology, Guangzhou, China; ^3^Cardiovascular Medicine Department, The Eighth Affiliated Hospital of Sun Yat-sen University, Shenzhen, China; ^4^Department of Medical Research Center, Sun Yat-sen Memorial Hospital, Sun Yat-sen University, Guangzhou, China

**Keywords:** idiopathic dilated cardiomyopathy, heart failure, reverse remodeling, predictive model, machine learning

## Abstract

**Introduction:** Left ventricular reverse remodeling (LVRR) is associated with decreased cardiovascular mortality and improved cardiac survival and also crucial for therapeutic options. However, there is a lack of an early prediction model of LVRR in first-diagnosed dilated cardiomyopathy.

**Methods:** This single-center study included 104 patients with idiopathic DCM. We defined LVRR as an absolute increase in left ventricular ejection fraction (LVEF) from >10% to a final value >35% and a decrease in left ventricular end-diastolic diameter (LVDd) >10%. Analysis features included demographic characteristics, comorbidities, physical sign, biochemistry data, echocardiography, electrocardiogram, Holter monitoring, and medication. Logistic regression, random forests, and extreme gradient boosting (XGBoost) were, respectively, implemented in a 10-fold cross-validated model to discriminate LVRR and non-LVRR, with receiver operating characteristic (ROC) curves and calibration plot for performance evaluation.

**Results:** LVRR occurred in 47 (45.2%) patients after optimal medical treatment. Cystatin C, right ventricular end-diastolic dimension, high-density lipoprotein cholesterol (HDL-C), left atrial dimension, left ventricular posterior wall dimension, systolic blood pressure, severe mitral regurgitation, eGFR, and NYHA classification were included in XGBoost, which reached higher AU-ROC compared with logistic regression (AU-ROC, 0.8205 vs. 0.5909, *p* = 0.0119). Ablation analysis revealed that cystatin C, right ventricular end-diastolic dimension, and HDL-C made the largest contributions to the model.

**Conclusion:** Tree-based models like XGBoost were able to early differentiate LVRR and non-LVRR in patients with first-diagnosed DCM before drug therapy, facilitating disease management and invasive therapy selection. A multicenter prospective study is necessary for further validation.

**Clinical Trial Registration:**http://www.chictr.org.cn/usercenter.aspx (ChiCTR2000034128).

## Introduction

Dilated cardiomyopathy (DCM) is the third leading cause of heart failure with decreased ejection fraction and the most important cause of heart transplantation (1, 2). Its 1-year mortality rate is as high as 25–30%, and its 5-year survival rate is <50% (3). Significant improvements in left ventricular end-diastolic diameter (LVDd) and left ventricular ejection fraction (LVEF) are referred to as left ventricular reverse remodeling (LVRR) (4). Despite the use of angiotensin-converting enzyme inhibitors (ACEIs), β-blocker, and mineralocorticoid receptor antagonists, LVRR happened only in approximately 37–52% of DCM patients (5–10). Therapy-induced LVRR has become an important prognostic tool in the management of patients with DCM (5, 11). If a patient is not responsive to medication, not only an early implantable cardioverter defibrillator may be necessary but also the timing of device therapy and insertion in the transplant list are important considerations since these aspects differ from those who are responsive to medication. Despite an increasing understanding of the progression of DCM, prognostic stratification of patients with early phases of DCM remains a challenge (12). It can be seen that early prediction of LVRR will help us to achieve precise management of patients with DCM.

Several early studies have reported the association between some clinical indexes and LVRR in DCM. Kawai et al. (13) first demonstrated that higher systolic blood pressure and lower pulmonary arterial wedge pressure at diagnosis were predictors of LVRR with medical therapy. Afterward, cardiac magnetic resonance was used for the prediction of LVRR. Several studies reported that late gadolinium enhancement at baseline provides a better prediction of LVRR (10, 14–17). However, there is no definite agreement in previous studies in regard to late gadolinium enhancement as an early predictor of LVRR (18). Genotype is also proven to associate with LVRR in DCM. It is reported that an inverse and independent association exists between structural cytoskeleton Z-disk gene rare variants and LVRR (19). Verdonschot et al. (7) also demonstrated that the model including mutation status performs better than the model with only clinical parameters (AUC = 0.760 vs. 0.742, *p* = 0.008). However, the difficult and expensive measurement limits their clinical application. Ruiz-Zamora et al. (20) found a simple logistic model including five variables with an AUC of 0.83. However, this model included several variables obtained at the end of follow-up, so we cannot make an early prediction for LVRR, which usually happens within 1 to 2 years in patients with DCM. Therefore, if we can identify LVRR in DCM when first diagnosed with a combination of several usual clinical parameters, it could help to make important clinical decisions concerning the need and timing of some therapies in patients with DCM.

Machine learning performs more objectively in selecting predictor variables and handles possible non-linear effects of variables better than traditional statistical methods. A tree-based ensemble algorithm can aggregate multiple weak learners to attain a stronger ensemble model by bagging and boosting two different ensemble ways, among which random forests and extreme gradient boosting (XGBoost) are, respectively, their representative methods. Random forests can use the bootstrap sampling method for avoiding instability of the model, while XGBoost algorithm was developed mainly for penalizing the structure of a decision tree to avoid overfitting (21). It has been found that this XGBoost technique outperforms other machine learning and deep learning methods in many competitions such as Kaggle and KDDCup (22). It has been successfully applied in numerous bioinformatics studies (23, 24) and medical studies (25, 26). Therefore, we conducted a retrospective real-world study and analyzed clinical data by using tree-based learning algorithms to build a predictive model and validate it.

## Materials and Methods

### Study Population

This study was a single-center real-world study. The clinical data of patients were collected from consecutively admitted patients with their first diagnosis of DCM at the Sun Yat-sen Memorial Hospital of Sun Yat-sen University between January 2014 and December 2017, and each of the patients had several follow-up records. DCM was diagnosed in keeping with the Chinese guidelines for the diagnosis and treatment of DCM (27) as follows: (1) LVDd >5.0 cm (female) or LVDd >5.5 cm (male); (2) LVEF <45% and left ventricular shortening fraction <25%; and (3) exclusion of valvular heart disease, congenital heart disease, ischemic heart disease, tachycardiomyopathy, and secondary DCM caused by systemic diseases. Patients with any of the following conditions were excluded: (1) alcoholic cardiomyopathy, peripartum cardiomyopathy, and other acquired DCM; (2) a history of HF treatment including ACEIs/angiotensin receptor blockers (ARBs)/angiotensin receptor-neprilysin inhibitors (ARNIs), adrenergic beta-receptor blockers, and mineralocorticoid receptor antagonists; (3) coronary heart disease (having narrowed coronary arteries 50% or more according to coronary angiography or coronary CTA), pulmonary heart disease, organic heart valvular disease, congenital heart disease, hypertensive heart disease, or pericardial disease; (4) not receiving medical therapy recommended by the Chinese Guidelines for the Diagnosis and Treatment of Heart Failure 2018 (28); (5) systemic diseases that may affect the structure and function of the heart, such as hyperthyroidism, hypothyroidism, amyloidosis, pheochromocytoma, systemic lupus erythematosus, or Behcet's disease; (6) cancer, severe infection, or severe renal dysfunction (estimated glomerular filtration rate (eGFR) <15 ml min^−1^·1.73 m^−2^); and (7) receiving cardiac resynchronization therapy or left ventricular assist device during follow-up. This study was approved by the institutional review board of Sun Yat-sen Memory Hospital and had therefore been performed in accordance with the ethical standards laid down in the 1964 Declaration of Helsinki and its later amendments. No informed consent was required because the data in our study were anonymized. All patients received standard medical therapy according to current guidelines (27, 28).

### Data Collection

All data of baselines and return visits were obtained from electronic health records including demographic characteristics, physical sign, comorbidities, laboratory indicators, electrocardiogram, 24-h dynamic electrocardiogram, echocardiographic data, and medication. The blood samples were collected after fasting for 12 h overnight. LVEF was measured using the apical biplane method and transthoracic echocardiography was performed as recommended by the American Society of Echocardiography (29) by a senior echocardiographer at admission and during the follow-up period. The New York Heart Association (NYHA) class was evaluated in this study within the first 8 h of admission.

### Definition of Variables

According to the European Association of Cardiovascular Imaging and the American Society of Echocardiography (30), the relative wall thickness was calculated as the ratio of two times the posterior wall thickness to LVDd. Left ventricular mass (LVM) was calculated according to the formula in (1). The normalization of LVM for body surface area was regarded as the left ventricular mass index. Body surface area was estimated by the formula in (2) (31). The eGFR was calculated using the modification of diet in renal disease equation (32). The doses of ACEIs/ARBs/ARNIs and β-blockers were evaluated by the ratio of the practical dose and target dose of certain drugs within 6 months (28).

(1) $$\begin{array}{l} LVM(g)=0.8\times 1.04\times [{\left(LVDd(cm)+LVPWd+IVSd\right)}^{3}\\ -LVDd^{3}]+0.6 \end{array}$$

(2) $$\begin{array}{rc} Bodysurfacearea\left(m^{2}\right)=0.007184\times height{\left(cm\right)}^{0.725} & \times weight{\left(kg\right)}^{0.425} \end{array}$$

### Return Visits

**Figure 1:**
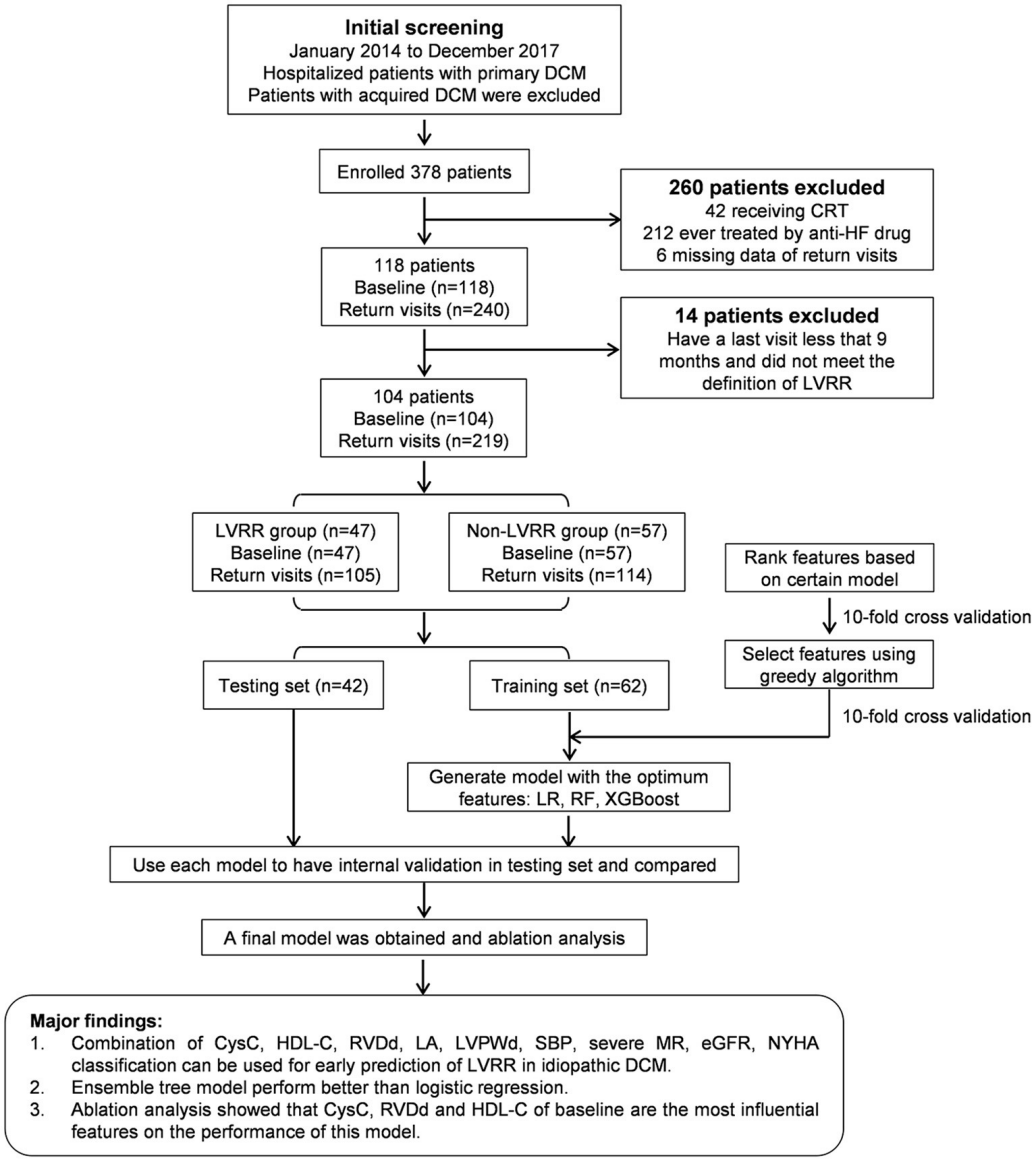
Overall flowchart and main results of this study. CRT, cardiac resynchronization therapy; CysC, cystatin C; DCM, dilated cardiomyopathy; eGFR, estimated glomerular filtration rate; HDL-C, high-density lipoprotein cholesterol; HF, heart failure; LA, left atrial dimension; LVDd, left ventricular end-diastolic diameter; LVEF, left ventricular ejection fraction; LVPWd, left ventricular posterior wall dimension; LVRR, left ventricular reverse remodeling; LR, logistic regression; MR, mitral regurgitation; NYHA, New York Heart Association; RF, random forest; RVDd, right ventricular end-diastolic dimension; SBP, systolic blood pressure; XGBoost, extreme gradient boosting.

The patients underwent a return visit as required. The end of visits was December 2018, the date of death or heart transplantation. Transthoracic echocardiography was performed in all visits. LVRR was defined as an absolute increase in LVEF from >10% to a final value >35% accompanied by a decrease in LVDd ≥10% (10) as assessed at any one visit and lasted until the last visit (median time 24 months, IQR 15–31). Non-LVRR was defined as an absolute increase in LVEF <10% or final value <35% or a decrease in LVDd <10% as assessed at all visits, except those in <9 months. Patients who did not meet the definition of LVRR and have a last visit <9 months were excluded (Figure 1).

### Statistical Analysis

Normally distributed variables are presented as the means ± standard deviations, while non-normally distributed variables are presented as medians with interquartile ranges. NT-proBNP, cTNT, D-dimer, and hsCRP were logarithmically transformed to approximate a normal distribution. The Levene test was used to explore the homogeneity of variance, and a *p*-value of <0.1 was considered to indicate heterogeneity of variance. Differences between groups were tested by the independent *t*-test or Mann–Whitney *U*-test for continuous variables and the chi-square test for categorical variables. De long test was used to detect if the difference between AUCs was statistically significant. Statistical significance was defined as a two-sided *p*-value of <0.05.

### Data Imputation

A total of 102 features were included for analysis and are described in Supplementary Table 1. Moreover, 65 variables had no missing data, 23 variables had <10% missing data, and the remaining 14 variables had >10% missing data. None of the variables had >50% missing data. All variables were standardized when selecting features and building models to mitigate the effect of the differences in dimensions between variables. The specific method is described in (3), where *X*_*k*0_ and *X*_*k*_ are the *k*th values of a certain variable before and after standardization, while *X*_min_ and *X*_max_ are the minimum and maximum values of a certain variable, respectively. *K*-nearest neighbors were used for the imputation of continuous and discrete variables, which took the average of *K* samples nearest to the missed point as its value.

(3) $$\begin{array}{r} X_{k}=\frac{X_{k0}{-X}_{\min}}{X_{\max}{-X}_{\min}} \end{array}$$

### Model Development

We chose three standard supervised machine learning methods for our data: XGBoost (21), random forest (33), and logistic regression with l_1_ penalty (34). The cases and controls involved in this study were randomly divided into training and testing sets with the ratio, train:test = 6:4. These models were trained on the training set with 10-fold cross-validation and were validated on the testing set (Figure 1). A grid search scheme was performed on the training set through the 10-fold cross-validation to search for the optimal combination of parameters of the model, where the training set was randomly split into 10 subsets. For each combination of parameters, nine subsets were trained for a model and the remaining one was used for validation of the model. The process was repeated for 10 times so that each subset was tested once and the average of their results was collected to measure the performance of the parameter combinations. As a result, we selected the parameter combination that reached the highest AUC to train a model based on the whole training set, and then the model was tested on the independent test set. The discrimination of models was evaluated using the receiver operating characteristic (ROC) curve. The calibration was performed using the isotonic regression (35) and evaluated by a calibration plot.

### Feature Selection

The distribution of each feature is shown in Supplementary Figure 1. Feature selection was also performed to optimize the feature combination in constructing a prediction model. In this study, we used a greedy feature selection algorithm based on the important features recommended by a specific model.

In general, a specific model was first pretrained to obtain the important features with 10-fold cross-validation on the training set, from which we select the feature greedily according to AUC. The important features included the features with an importance greater than zero. In the greedy searching process, the selection algorithm began with an empty set of features and iteratively searched the best feature from the remaining feature set and added the best feature to the empty set for a higher AUC. This procedure was repeated until the remaining feature set was empty or AUC no longer increased, leading to a best feature subset for building a final prediction model.

### Machine Learning and Statistical Tools

The research data of our study were assessed with the machine learning tools of the scikit-learn project. The tool environment we applied was Python 3.7.6 with scikit-learn 0.22 running on Anaconda 3 (4.8.5-Linux-x86_64) for data processing, modeling, and evaluation. SPSS version 22.0 (IBM SPSS Statistics, IBM Corporation, Armonk, NY, USA) was used to perform the descriptive statistics.

## Results

### Baseline Characteristics

A total of 378 inpatient clinical data points from 104 patients were collected. Among the 104 patients analyzed, LVRR was observed in 47 individuals (45.2%) (Figure 1). The characteristics and the distribution of the patients are shown in Table 1 and Supplementary Figure 1. Patients who developed LVRR were more likely to have a higher systolic blood pressure, higher platelet count, lower serum D-dimer level, higher high-density lipoprotein cholesterol (HDL-C) level, smaller left atrial dimension, and smaller right ventricular end-diastolic dimension and were less likely to suffer from severe mitral regurgitation (MR). The use or doses of ACEIs/ARBs/ARNIs and β-blockers were not significantly different between the two groups.

**Table 1:** Characteristics of patients grouped by left ventricular reverse remodeling.

**Variables**	**LVRR (*n* = 47)**	**Non-LVRR (*n* = 57)**	***p-*value**
Age (years)	54.7 ± 15.3	55.1 ± 14.0	0.899
Female, *n* (%)	12 (25.5)	14 (24.6)	0.909
Body mass index (kg/m^2^)	(38)^a^ 24.67 ± 4.74	(52) 25.05 ± 4.25	0.692
SBP (mmHg)	130.2 ± 19.3	120.7 ± 20.8	^*^0.016
DBP (mmHg)	83.6 ± 16.6	79.0 ± 13.5	0.125
Heart rate (/min)	91.3 ± 16.3	87.0 ± 17.2	0.199
NYHA class			^*^0.042
I, *n* (%)	4 (8.5)	2 (3.5)	
II, *n* (%)	15 (31.9)	10 (17.5)	
III, *n* (%)	22 (46.8)	34 (59.6)	
IV, *n* (%)	6 (12.8)	11 (19.3)	
Smoking, *n* (%)	20 (42.6)	22 (38.6)	0.682
Drinking, *n* (%)	6 (12.8)	12 (21.1)	0.266
Hypertension, *n* (%)	18 (38.3)	18 (31.6)	0.473
Diabetes, *n* (%)	8 (17.0)	7 (12.3)	0.493
Atrial fibrillation, *n* (%)	5 (10.6)	14 (24.6)	0.067
VT or VF, *n* (%)	2 (4.3)	4 (7.0)	0.858
Atrioventricular block, *n* (%)	6 (12.8)	8 (14.0)	0.850
ICD, *n* (%)	31 (66.0)	29 (50.9)	0.121
**Laboratory values**			
White blood cell (×10^9^/L)	7.81 (6.28–10.01)	7.42 (6.12–8.78)	0.376
Hemoglobin (g/L)	137.1 ± 19.0	138.9 ± 16.4	0.613
Platelet (×10^9^/L)	251.1 ± 84.4	209.3 ± 53.5	0.004^**^
Lymphocyte (%)	24.1 ± 7.5	24.7 ± 9.7	0.718
Lymphocyte (×10^9^/L)	1.92 ± 0.51	1.86 ± 0.71	0.611
Neutrophils (%)	66.8 ± 8.5	67.0 ± 10.0	0.908
Neutrophils (×10^9^/L)	5.84 ± 2.69	5.52 ± 2.62	0.532
Mononuclear cell (%)	6.4 ± 2.2	6.0 ± 2.2	0.350
Mononuclear cell (×10^9^/L)	0.553 ± 0.326	0.474 ± 0.209	0.156
RDW-CV (%)	0.14 ± 0.02	0.14 ± 0.02	0.390
Prothrombin activity (%)	(46) 78.5 ± 19.7	69.0 ± 23.5	0.032^*^
Fibrinogen (g/L)	(46) 3.28 ± 1.06	2.98 ± 0.91	0.124
Prothrombin time (s)	(46) 12.2 (11.4–12.9)	12.7 (11.7–14.5)	0.034^*^
APTT (s)	(46) 27.7 (25.1–31.6)	28.9 (26.1–31.8)	0.403
International normalized ratio	(46) 1.08 (1.00–1.14)	1.11 (1.02–1.25)	0.053
lg D-dimer (mg/L)	(45) −0.36 ± 0.51	−0.09 ± 0.50	0.007^**^
lg NT-proBNP (pg/ml)	3.28 ± 0.51	(56) 3.41 ± 0.52	0.191
lg CTNT (pg/ml)	(43) 1.25 ± 0.41	(46) 1.38 ± 0.41	0.130
Creatine kinase (U/L)	66 (46–101)	83 (52–140)	0.153
Creatine kinase MB (U/L)	11 (9–14)	13 (10–16)	0.106
ALT (U/L)	25.0 (15.0–49.0)	(56) 29.0 (18.0–51.8)	0.193
AST (U/L)	23.0 (20.0–39.0)	(56) 29.5 (21.0–45.3)	0.138
γ-Glutamyltransferase (U/L)	(46) 44.5 (20.8–94.0)	(55) 58.0 (30.0–97.0)	0.417
FBG (mmol/L)	4.8 (4.3–5.7)	(56) 4.9 (4.4–5.6)	0.538
Cystatin C (mg/L)	(27) 0.94 ± 0.22	(40) 1.06 ± 0.30	0.084
Urea (mmol/L)	5.7 (4.7–7.8)	6.7 (5.6–8.1)	0.087
CO_2_CP (mmol/L)	25.3 ± 4.8	24.9 ± 3.5	0.576
eGFR (ml·min^−1^·1.73 m^−2^)	78.87 ± 46.58	66.18 ± 17.02	0.059
Uric acid (μmol/L)	479.1 ± 170.6	(56) 546.6 ± 178.4	0.054
Triglyceride (mmol/L)	1.08 (0.88–1.57)	(55) 1.28 (0.87–1.57)	0.692
Total cholesterol (mmol/L)	4.49 ± 0.89	(55) 4.44 ± 1.57	0.861
LDL-C (mmol/L)	2.86 ± 0.67	(55) 2.81 ± 0.82	0.743
HDL-C (mmol/L)	1.10 ± 0.32	(55) 0.93 ± 0.26	0.005^**^
Albumin (g/L)	(46) 37.0 ± 4.8	(55) 36.0 ± 5.1	0.319
lg hsCRP (mg/L)	(45) 0.60 ± 0.76	(54) 0.69 ± 0.64	0.491
Hemoglobin A1c (%)	(35) 6.04 ± 0.65	(43) 6.15 ± 1.07	0.563
Free T3 (pmol/L)	(41) 4.88 ± 1.36	(49) 4.58 ± 0.97	0.235
Free T4 (pmol/L)	(41) 18.75 ± 4.51	(49) 18.04 ± 3.31	0.393
TSH (mIU/L)	(42) 1.43 (0.98–2.68)	(49) 1.66 (0.93–3.11)	0.558
Superoxide dismutase (U/L)	(45) 123.4 ± 17.4	(54) 121.0 ± 18.9	0.516
Adenylic deaminase (U/L)	(31) 10.00 ± 2.53	(45) 11.29 ± 3.87	0.084
Free fatty acid (μmol/L)	(44) 556.8 ± 243.8	(54) 706.9 ± 346.0	0.014^**^
K (mmol/L)	3.86 ± 0.37	3.93 ± 0.43	0.403
Na (mmol/L)	140.52 ± 2.94	139.59 ± 2.92	0.111
Cl (mmol/L)	104.0 ± 3.7	103.4 ± 3.3	0.446
Ca (mmol/L)	2.20 ± 0.12	2.19 ± 0.10	0.686
P (mmol/L)	(44) 1.24 ± 0.20	(53) 1.26 ± 0.36	0.738
**Electrocardiograph**			
PR interval (ms)	(40) 163.3 ± 35.2	(43) 161.2 ± 40.0	0.802
QRS interval (ms)	(36) 110.8 ± 32.9	(49) 110.3 ± 33.5	0.945
QTc interval (ms)	(44) 442.6 ± 82.2	(54) 434.1 ± 51.4	0.532
Left bundle branch block	11 (23.4)	5 (8.8)	0.040^*^
**Holter**			
Number of VPB	(28) 54 (8–1,126)	(38) 657 (58–1,995)	0.066
Number of APB	(28) 20 (6–45)	(38) 15 (0–55)	0.490
**Echocardiography**			
LVEF (%)	30.2 ± 5.8	30.2 ± 6.9	0.963
LVDd (mm)	69.0 ± 8.6	67.2 ± 8.4	0.282
AOR (mm)	21.8 ± 2.1	21.3 ± 1.7	0.174
LA (mm)	41.3 ± 6.7	44.3 ± 6.2	0.020^*^
RVDd (mm)	21.7 ± 3.9	23.8 ± 4.3	0.011^*^
IVSd (mm)	9.3 ± 1.7	9.3 ± 1.9	0.905
LVPWd (mm)	9.4 ± 1.7	9.3 ± 1.9	0.795
LVMI (g/m^2^)	(38) 173.8 ± 44.5	(52) 160.5 ± 43.5	0.147
RWT	0.28 ± 0.06	0.29 ± 0.08	0.535
**Mitral regurgitation**			
Severe, *n* (%)	10 (21.3)	23 (40.4)	0.038^*^
**Tricuspid regurgitation**			
Moderate and severe, *n* (%)	11 (23.4)	25 (43.9)	0.029^*^
**Medication**			
ACEI/ARB/ARNI (%)	43 (91.5)	48 (84.2)	0.264
ACEI/ARB/ARNI doses (%)	0.50 (0.50–1.00)	0.50 (0.33–1.00)	0.301
Increasing doses of ACEI/ARB/ARNI (%)	3 (6.4)	2 (3.5)	0.825
β-Blocker	37 (78.7)	44 (77.2)	0.852
β-Blocker doses	0.20 (0.06–0.25)	0.13 (0.06–0.25)	0.371
Increasing doses of β-blocker	8 (17.0)	6 (10.5)	0.334
MRA	44 (93.6)	54 (94.7)	>0.999
Diuretic	45 (95.7)	57 (100)	0.202
Digoxin	37 (78.7)	45 (78.9)	0.978
Statin	14 (29.8)	16 (28.1)	0.847
Anticoagulation	2 (4.3)	10 (17.5)	0.035^*^
Antiplatelet	7 (14.9)	10 (17.5)	0.716
Amiodarone	7 (14.9)	7 (12.3)	0.698
Trimetazidine	8 (17.0)	21 (36.8)	0.025^*^
Ivabradine	0 (0)	3 (5.3)	0.314

*ACEIs, angiotensin-converting enzyme inhibitors; ALT, alanine aminotransferase; AOR, aortic root diameter; APB, atrial premature beat; APTT, activated partial thromboplastin time; ARBs, angiotensin receptor blockers; ARNIs, angiotensin receptor-neprilysin inhibitors; AST, aspartate aminotransferase; CO_2_CP, carbon dioxide combining power; DBP, diastolic blood pressure; eGFR, estimated glomerular filtration rate; FBG, fasting blood glucose; HDL-C, high-density lipoprotein cholesterol; ICD, implantable cardioverter defibrillator; IVSD, interventricular septal dimension; LA, left atrial; LDL-C, low-density lipoprotein cholesterol; LVDd, left ventricular end-diastolic dimension; LVEF, left ventricular ejection fraction; LVMI, left ventricular mass index; LVPWd, left ventricular posterior wall dimension; LVRR, left ventricular reverse remodeling; MRA, mineralocorticoid receptor antagonist; NYHA, New York Heart Association; RDW-CV, red cell distribution width variable coefficient; RWT, relative wall thickness; RVDd, right ventricular end-diastolic dimension; SBP, systolic blood pressure; TSH, thyroid stimulating hormone; VF, ventricular fibrillation; VPB, ventricular premature beat; VT, ventricular tachycardia*.

a *The remaining valid data regardless of the missing data*.

* *p < 0.05*,

** *p < 0.01*.

### Data From Visits

**Figure 2:**
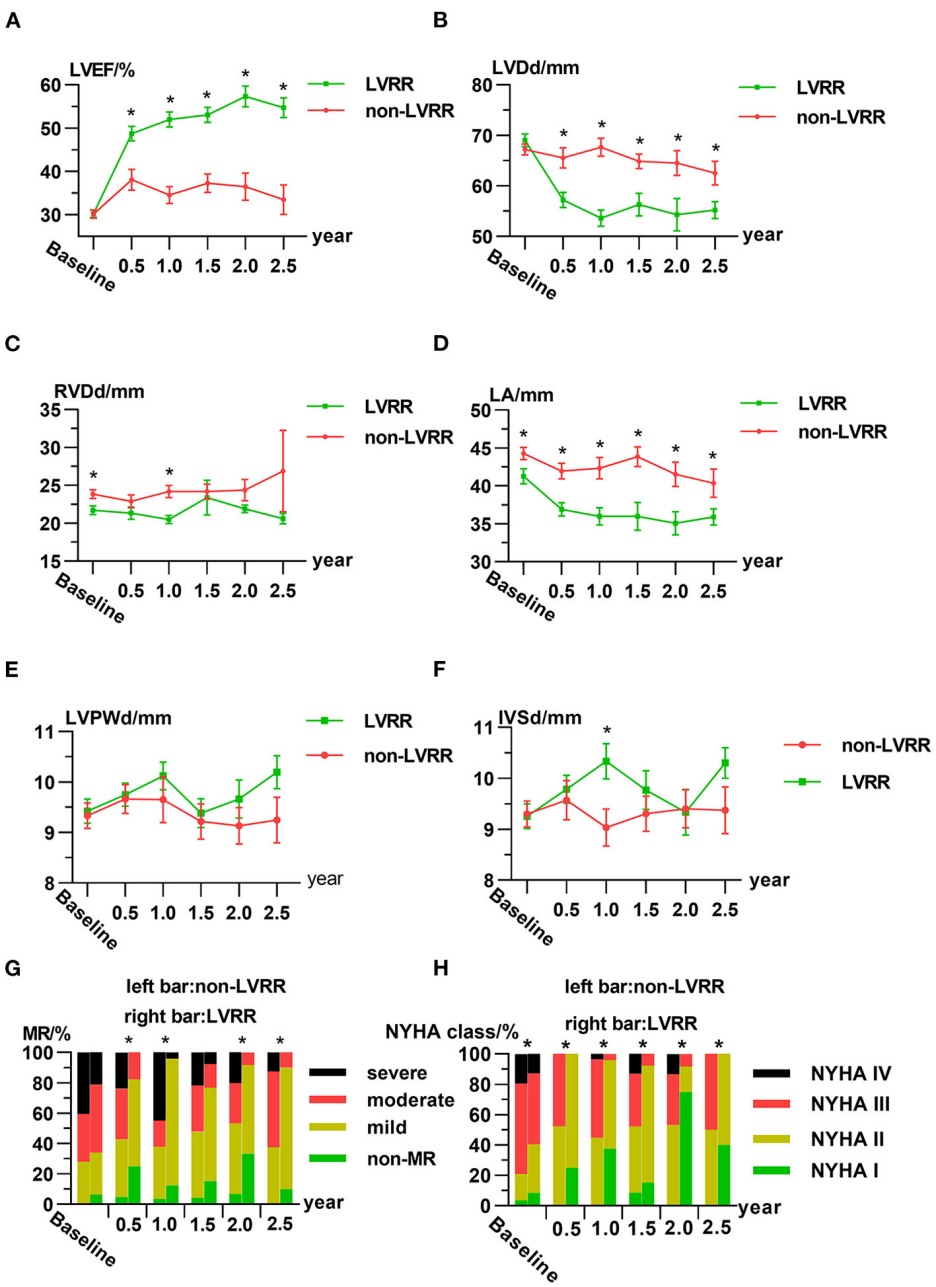
Characteristics of the LVRR group and non-LVRR group at the first visit and return visits. Line chart for the averages of **(A)** LVEF, **(B)** LVDd, **(C)** RVDd, **(D)** LA, **(E)** LVPWd, and **(F)** IVSd. **(G,H)** Ratio of the severity of MR and NYHA functional class over time. The data are presented as the mean ± standard error **(A–F)**. In **(A–F)**, *p ≤ 0.05 by non-paired Student's *t*-test between two groups. In **(G)**, *p ≤ 0.05 comparing the percentage of patients who are above moderate or severe in both groups by chi-square test. In **(H)**, *p ≤ 0.05 by Mann–Whitney *U*-test. IVSd, interventricular septal dimension; LA, left atrial dimension; LVDd, left ventricular end-diastolic dimension; LVEF, left ventricular ejection fraction; LVPWd, left ventricular posterior wall dimension; LVRR, left ventricular reverse remodeling; MR, mitral regurgitation; NS, no statistically significant difference; NYHA, New York Heart Association; RVDd, right ventricular end-diastolic dimension.

All patients completed return visits. The details of the time distributions of visits are shown in Supplementary Figure 2. LVEF and LVDd were similar between the two groups at baselines, but in the LVRR group, LVEF, LVDd, left atrial dimension, and severity of MR were improved significantly and tended to be stable after 1 year (Figures 2A,B,D,G). Right ventricular end-diastolic dimension, left ventricular posterior wall dimension, and interventricular septal dimension showed no obvious change during return visits both in LVRR and non-LVRR groups (Figures 2C,E,F). NYHA functional class in the LVRR group was better than that in non-LVRR groups at each time point (Figure 2H).

### Classifier Model Development and Validation

**Figure 3:**
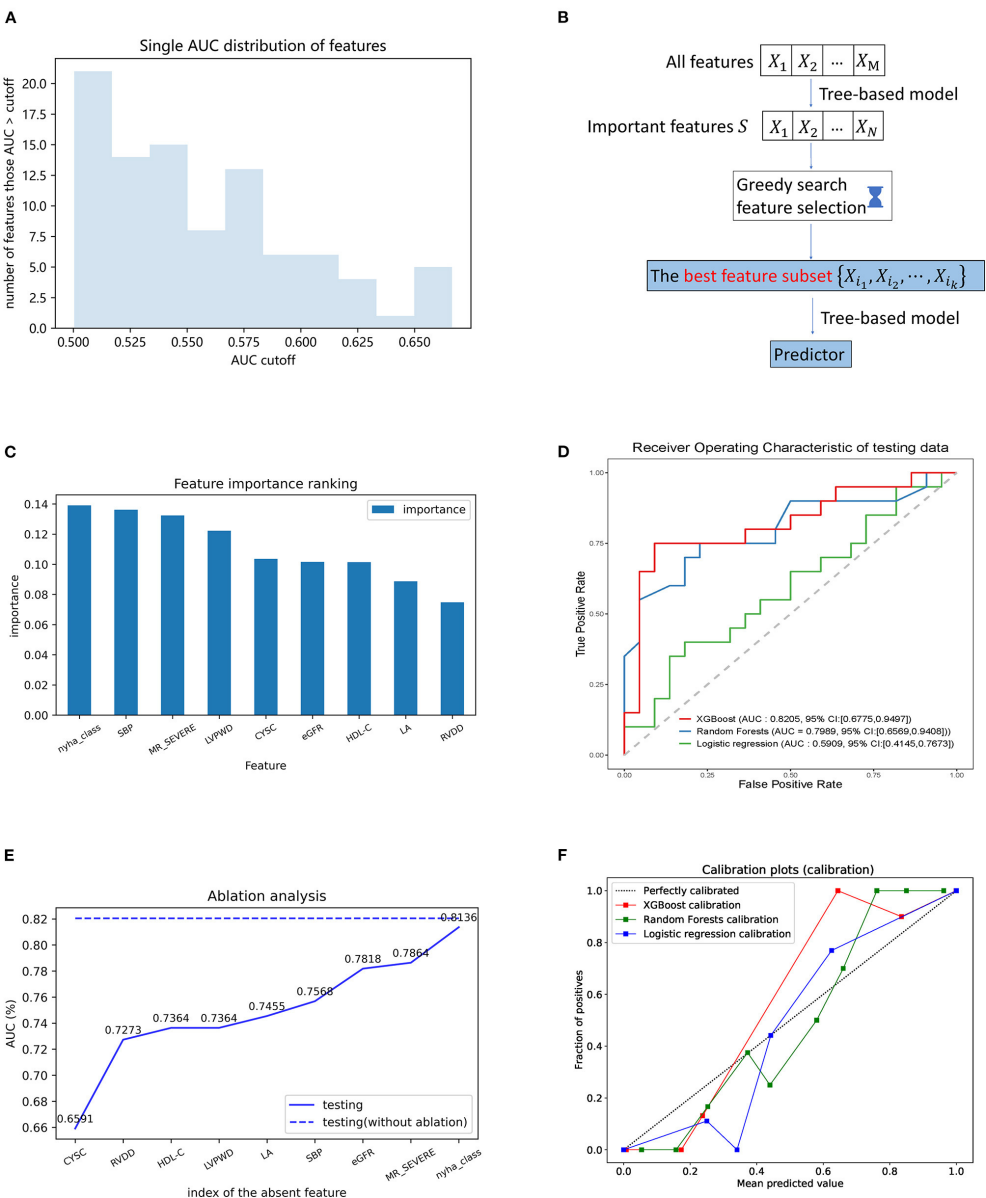
Building model procedure and contributions of the selected features in the prediction. **(A)** AUC distribution obtained by individual features in the prediction of LVRR and non-LVRR; **(B)** flowchart of greedy feature selection by XGBoost; **(C)** greedy feature selection provides the nine best features and the comparison of their importance; **(D)** receiver operating characteristic curve of three models in testing set. Green, blue, and red curves were generated by the logistic regression, the random forest, and the XGBoost algorithms, respectively; **(E)** ablation analysis is performed to evaluate the contributions of each feature in the prediction; **(F)** calibration plot of three models. Blue, green, and red curves were generated by the logistic regression, the random forest, and the XGBoost algorithms, respectively. CysC, cystatin C; eGFR, estimated glomerular filtration rate; HDL-C, high-density lipoprotein cholesterol; LA, left atrial; LVPWd, left ventricular posterior wall dimension; MR, mitral regurgitation; NYHA, New York Heart Association; RVDd, right ventricular end-diastolic dimension; SBP, systolic blood pressure.

The individual features were tested in their ability to classify the LVRR and the non-LVRR. As indicated by Figure 3A, there are more than 20 features (30.12%) with an AUC that only reached slightly more than 0.5, and only five features with an AUC larger than 0.65. The maximum AUC of all features is <0.7. Thus, it is necessary to identify the combined effects of the features in discriminating the LVRR and the non-LVRR.

The feature selection procedure is shown in Figure 3B. The tree-based model was first pretrained on the training set to obtain the important features (we describe the result of XGBoost here). Finally, 33 features were selected as important. From these features, we used greedy search to obtain the feature subset which can reach an accurate classification result. The greedy searching provided nine features. Figure 3C shows their importance rank. These features were used to train an XGBoost model with 10-fold cross-validation, which consequently achieved AUC 0.8463 and 0.8205 on the CV (cross-validation) set and test set, respectively (Figure 3D and Supplementary Figure 3). The similarity of the AUC on training and testing set also accounts for the robustness of the model.

Ablation analysis was performed with 10-fold cross-validation to estimate the contributions of each feature in the prediction. As shown in Figure 3E, the absence of each of them could cause a decline of the AUC. Moreover, we observed that cystatin C is the most important feature above all. The ablation of cystatin C can reduce the AUC from 0.8205 to 0.6591.

**Figure 4:**
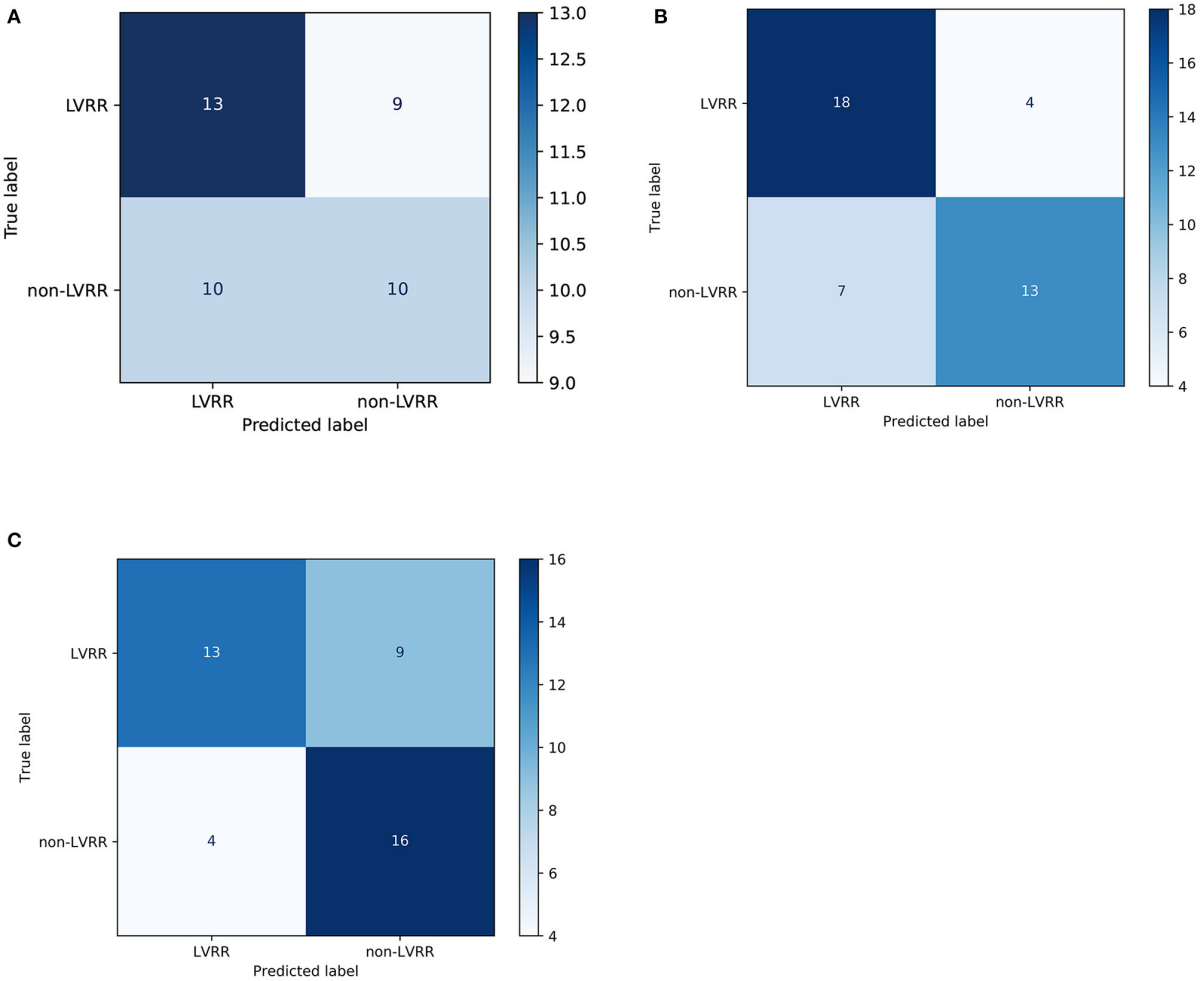
Confusion matrices of the predictive models. The confusion matrix of the logistic regression model **(A)**, random forest **(B)**, and XGBoost **(C)** in the testing set (~40% of the cohort). Predicted label: the sum of each column represents the predicted sample number of the classes. True label: the sum of each row represents the true sample number of the classes.

By comparison, we tested other machine learning methods including logistic regression with l_1_ penalty and random forests with the same process shown in Figure 3B. As shown in Figure 3D, our method using XGBoost and random forests achieved better AUCs than the linear model on the test set, with AUCs of 0.8205 (95% CI 0.6775–0.9497, *p* = 0.0119 vs. LR) and 0.7989 (95% CI 0.6589–0.9408, *p* = 0.0258 vs. LR), respectively. From the confusion matrix of each model shown in Figure 4, we found that the XGBoost can correctly classify 13 of 22 LVRR patients and 16 of 20 non-LVRR patients on the test set, while the random forests can correctly classify 18 of 22 LVRR patients and 13 of 20 non-LVRR patients. The above fact indicated that XGBoost and random forests showed different advantages in classifying the non-LVRR patients and LVRR patients. Moreover, these two tree-based models are both superior to the logistic regression model in classifying LVRR and non-LVRR. Table 2 also reveals the truth by comparing the recall and the sensitivity measurements in classifying LVRR and non-LVRR. Furthermore, we did calibration analysis of the above three models in order to get more statistic evidence for model performance comparison. As shown in Figure 3F, these models had similar calibration.

**Table 2:** Comparison of model performance.

**Models**	**Classification**	**Precision**	**Recall**	**F1 score**
Random forest	Non-LVRR	0.7647	0.5909	0.7027
	LVRR	0.72	0.8182	0.7660
XGBoost	Non-LVRR	0.64	0.8	0.7111
	LVRR	0.7647	0.5909	0.6667
Logistic regression	Non-LVRR	0.5263	0.5	0.5128
	LVRR	0.5652	0.5909	0.5778

*LVRR, left ventricular reverse remodeling*.

## Discussion

In this study, our key findings are as follows: (1) the XGBoost and random forest classifiers combining routine clinical indexes collected before treatment show higher accuracy than logistic regression in predicting LVRR in patients with DCM. (2) Baseline cystatin C, right ventricular end-diastolic dimension, and HDL-C are the most important features in this model, but not LVEF and LVDd. These machine classifiers might be useful to identify the patients who may not respond to the medication and in whom early clinical monitoring and early implementation of preventive strategies may be helpful.

To the best of our knowledge, this is the first study using ensemble tree models of machine learning to predict LVRR. Compared with traditional regression, these models avoid presupposing a linear relation between different variables and the assumptions that are required for correctness of statistical models. In our study, optimized classifiers such as XGBoost and random forest performed with similar better accuracy in predicting LVRR. These ensemble tree models might be useful for improvement in risk factor management in DCM. Unlike the assessment for business risk or the prediction for mortality risk, we pay more attention to better discrimination in the early identification of non-LVRR in DCM, which may be followed more intensively. For the XGBoost model that performed more accurately in differentiating non-LVRR, it was chosen as the final model for subsequent analysis. Moreover, we also found that a single clinical index cannot predict LVRR well, which indicated that LVRR is a consequence of coaction of several factors. At last, we built the XGBoost model including four echocardiogram indexes, three routine laboratory indexes, systolic blood pressure, and NYHA functional class. LVRR is more likely to occur in patients with NYHA functional class I–II, compared with those with NYHA functional class III–IV [61.3% (19/31) vs. 38.4% (28/73), *p* = 0.032]. Patients with NYHA functional class I–II may be in the early stages of the disease. It has been reported that a shorter duration of disease is associated with a higher likelihood of recovery of LVEF (4). This result is also consistent with some prior reports (20, 36).

Our ablation analysis showed that serum cystatin C contributes remarkably for the predictive model, which is a similar finding to those of previous studies on prognosis of dilated cardiomyopathy. It has been reported that cystatin C was the best predictor of LVEF increase in DCM patients (37). Chatterjee et al. (38) revealed that baseline cystatin C showed incremental benefit in the prediction of cardiac resynchronization therapy non-response compared with conventional renal markers. As we all know, cystatin C is not subject to variability in renal filtration and is considered to be a more stable renal marker, which is less sensitive to gender and age. However, cystatin C may not only serve as a marker of intersecting cardio-renal pathways in patients with DCM but also associate with cathepsin B inhibition, collagen accumulation, and myocardial fibrosis, as an inhibitor of cathepsins, which play a role in the degradation of the extracellular matrix (39). It has been reported that an excess of cystatin C leads to extracellular tissue inhibitor of metalloproteinase-1 and osteopontin accumulation in human cardiac fibroblast cells (40). We speculate that cystatin C takes part in alterations in collagen metabolism and the process of cardiac fibrosis in DCM, which was shown as a key determinant of left ventricular remodeling in DCM (14). Hence, the combination of cystatin C and eGFR (calculated by creatinine) leads to obvious improvement in our model for LVRR in DCM.

In the ablation analysis, we can see that there are four important clinical indexes of cardiac structure obtained by echocardiography. Echocardiography represents the first-line examination in patients with DCM. Our results are similar to those of previous studies on prognosis and dilated cardiomyopathy. Barison et al. (41) reported that prognosis in patients with <35% LVEF was not significantly worse than those with LVEF >35% (*p* = 0.476). La Vecchia et al. (42) reported that right ventricular end-diastolic volume but not LVEF was demonstrated as an independent predictor of transplant-free survival. Recent studies also found that right ventricular function can be used for prediction in the prognosis of DCM (42, 43). Furthermore, baseline right ventricular dysfunction was proven as a stronger predictor than other known prognostic factors, such as NYHA functional class, functional mitral regurgitation (43), and systolic blood pressure (5, 13). Right ventricular dysfunction may reflect an increased pulmonary artery pressure (44), which may represent an advance stage of ventricular remodeling. Although, right ventricular end-diastolic dimension did not adequately reflect right ventricular function, the combination of adverse remodeling characteristics, such as functional mitral regurgitation and enlargement of other chambers, can provide valuable information for prediction.

HDL-C was another important variable that contributes much in a predictive model from ablation analysis. Emmens et al. (45) reported an inverse association between HDL-C and all-cause mortality or MACE in HFrEF, but not in HFpEF. Freitas et al. (46) also obtained a similar result. The mechanism underlying the association between HDL-C and left ventricular reverse remodeling is not yet clear. Emerging evidence shows that subfractions of HDL have antioxidant, anti-inflammatory, and endothelial cell protective capacity (47–49). Sampietro et al. (50) also found a significant association between HDL-C level and idiopathic DCM and a negative correlation between HDL-C level and inflammation markers, which are similar to our results (Supplementary Figure 4). It may be because serum NT-proBNP levels at first admission can indicate only a short congestive state (51), and there are several novel mechanisms between HDL-C level and left ventricular reverse remodeling in patients with DCM; in our study, there are obvious differences in the HDL-C level but not in hsCRP and NT-proBNP between the LVRR and non-LVRR groups. In addition, DCM is a kind of clinical syndrome which has an impact on multiple organ systems and diverse etiologies. We need the timely identification of LVRR, which can be helpful for their precise management. Machine learning applications might be an attractive option to provide a solution to this problem.

### Study Limitations

A limitation of our study is that it is a single-center and retrospective study, so we should obtain stronger evidence by performing a large sample prospective study and external validation. A further limitation is that we focused on the predictive performance rather than statistical inference. Therefore, we cannot draw a conclusion about risk factors. In addition, compared with the linear models, tree-based models usually own some unexplainable feature mechanism.

## Conclusions

XGBoost and random forest algorithms exhibit good performance for predicting LVRR in patients with DCM. The combination of routine laboratory indicators and echocardiography indexes can be used for predicting LVRR in DCM. These machine learning classifiers might be useful for accurate management and risk evaluation of patients with DCM.

## Data Availability Statement

The data used to support the findings of this study are available from the corresponding author upon reasonable request.

## Ethics Statement

The studies involving human participants were reviewed and approved by Medical Ethics Committee of Sun Yat-Sen Memorial Hospital. Written informed consent for participation was not required for this study in accordance with the national legislation and the institutional requirements.

## Author Contributions

JW, YZ, YC, and HZ contributed to the conception and design of the study. XX and MY contributed to the collection of data. XX, MY, HZ, and SX contributed to the analysis and interpretation of the data. XX, XW, YJ, ZL, and YZ contributed to the drafting of the article. All authors have revised the manuscript critically for important intellectual content, read, and approved the final manuscript.

## Conflict of Interest

The authors declare that the research was conducted in the absence of any commercial or financial relationships that could be construed as a potential conflict of interest.

## Publisher's Note

All claims expressed in this article are solely those of the authors and do not necessarily represent those of their affiliated organizations, or those of the publisher, the editors and the reviewers. Any product that may be evaluated in this article, or claim that may be made by its manufacturer, is not guaranteed or endorsed by the publisher.

## References

[1] Taylor DO Edwards LB Boucek MM Trulock EP Waltz DA Keck BM. Registry of the International society for heart and lung transplantation: twenty-third official adult heart transplantation report−2006. J Heart Lung Transplant. (2006) 25:869–79. 10.1016/j.healun.2006.05.002 16890107

[2] Song L. Clinical and pathological findings of 400 heart transplants in fuwai hospital. Chin Circ J. (2015) 30:204–5. 10.3969/j.issn.1000-3614.2015.03.002

[3] Dec GW Fuster V. Idiopathic dilated cardiomyopathy. N Engl J Med. (1994) 331:1564–75. 10.1056/NEJM199412083312307 7969328

[4] Wilcox J Fang J Margulies K Mann D. Heart failure with recovered left ventricular ejection fraction: JACC scientific expert panel. J Am Coll Cardiol. (2020) 76:719–34. 10.1016/j.jacc.2020.05.075 32762907

[5] Merlo M Pyxaras SA Pinamonti B Barbati G Di Lenarda A Sinagra G. Prevalence and prognostic significance of left ventricular reverse remodeling in dilated cardiomyopathy receiving tailored medical treatment. J Am Coll Cardiol. (2011) 57:1468–76. 10.1016/j.jacc.2010.11.030 21435516

[6] Choi JO Kim EY Lee GY Lee SC Park SW Kim DK. Predictors of left ventricular reverse remodeling and subsequent outcome in nonischemic dilated cardiomyopathy. Circ J. (2013) 77:462–9. 10.1253/circj.CJ-12-0507 23095684

[7] Verdonschot JAJ Hazebroek MR Wang P Wijk SSV Merken JJ Adriaansen YA. Clinical phenotype and genotype associations with improvement in left ventricular function in dilated cardiomyopathy. Circ Heart Fail. (2018) 11:e005220. 10.1161/CIRCHEARTFAILURE.118.005220 30571196

[8] Ikeda Y Inomata T Iida Y Iwamoto-Ishida M Nabeta T Ishii S. Time course of left ventricular reverse remodeling in response to pharmacotherapy: clinical implication for heart failure prognosis in patients with idiopathic dilated cardiomyopathy. Heart Vessels. (2016) 31:545–54. 10.1007/s00380-015-0648-2 25686768

[9] Hoshikawa E Matsumura Y Kubo T Okawa M Yamasaki N Kitaoka H. Effect of left ventricular reverse remodeling on long-term prognosis after therapy with angiotensin-converting enzyme inhibitors or angiotensin II receptor blockers and β blockers in patients with idiopathic dilated cardiomyopathy. Am J Cardiol. (2011) 107:1065–70. 10.1016/j.amjcard.2010.11.033 21296328

[10] Kubanek M Sramko M Maluskova J Kautznerova D Weichet J Lupinek P. Novel predictors of left ventricular reverse remodeling in individuals with recent-onset dilated cardiomyopathy. J Am Coll Cardiol. (2013) 61:54–63. 10.1016/j.jacc.2012.07.072 23287372

[11] McNamara DM Starling RC Cooper LT Boehmer JP Mather PJ Janosko KM. Clinical and demographic predictors of outcomes in recent onset dilated cardiomyopathy: results of the IMAC (intervention in myocarditis and acute cardiomyopathy)-2 study. J Am Coll Cardiol. (2011) 58:1112–8. 10.1016/j.jacc.2011.05.033 21884947 PMC6467576

[12] Merlo M Caiffa T Gobbo M Adamo L Sinagra G. Reverse remodeling in dilated cardiomyopathy: insights and future perspectives. Int J Cardiol Heart Vasc. (2018) 18:52–7. 10.1016/j.ijcha.2018.02.005 29876504 PMC5988485

[13] Kawai K Takaoka H Hata K Yokota Y Yokoyama M. Prevalence, predictors, and prognosis of reversal of maladaptive remodeling with intensive medical therapy in idiopathic dilated cardiomyopathy. Am J Cardiol. (1999) 84:671–6. 10.1016/S0002-9149(99)00414-2 10498137

[14] Masci PG Schuurman R Andrea B Ripoli A Coceani M Chiappino S. Myocardial fibrosis as a key determinant of left ventricular remodeling in idiopathic dilated cardiomyopathy: a contrast-enhanced cardiovascular magnetic study. Circ Cardiovasc Imaging. (2013) 6:790–9. 10.1161/CIRCIMAGING.113.000438 23934992

[15] Ikeda Y Inomata T Fujita T Iida Y Nabeta T Ishii S. Cardiac fibrosis detected by magnetic resonance imaging on predicting time course diversity of left ventricular reverse remodeling in patients with idiopathic dilated cardiomyopathy. Heart Vessels. (2016) 31:1817–25. 10.1007/s00380-016-0805-2 26843195

[16] Ishii S Inomata T Fujita T Iida Y Ikeda Y Nabeta T. Clinical significance of endomyocardial biopsy in conjunction with cardiac magnetic resonance imaging to predict left ventricular reverse remodeling in idiopathic dilated cardiomyopathy. Heart Vessels. (2016) 31:1960–8. 10.1007/s00380-016-0815-0 26920939

[17] Xu Y Li W Wan K Liang Y Jiang X Wang J. Myocardial tissue reverse remodeling after guideline-directed medical therapy in idiopathic dilated cardiomyopathy. Circ Heart Fail. (2021) 14:e007944. 10.1161/CIRCHEARTFAILURE.120.007944 33185117

[18] Tayal U Prasad SK. Myocardial remodelling and recovery in dilated cardiomyopathy. JRSM Cardiovasc Dis. (2017) 6:204800401773447. 10.1177/2048004017734476 29051817 PMC5637962

[19] Ferro MD Stolfo D Altinier A Gigli M Perrieri M Ramani F. Association between mutation status and left ventricular reverse remodelling in dilated cardiomyopathy. Heart. (2017) 103:1704–10. 10.1136/heartjnl-2016-311017 28416588

[20] Ruiz-Zamora I Rodriguez-Capitan J Guerrero-Molina A Morcillo-Hidalgo L Rodriguez-Bailon I Gomez-Doblas JJ. Incidence and prognosis implications of long term left ventricular reverse remodeling in patients with dilated cardiomyopathy. Int J Cardiol. (2016) 203:1114–21. 10.1016/j.ijcard.2015.11.099 26651150

[21] Chen T Guestrin C. Xgboost: a scalable tree boosting system. In: Proceedings of the 22nd ACM SIGKDD International Conference on Knowledge Discovery and Data Mining. San Francisco, CA: ACM (2016). p. 785–94. 10.1145/2939672.2939785

[22] Dhaliwal S Nahid AA Abbas R. Effective intrusion detection system using XGBoost. Information. (2018) 9:149. 10.3390/info9070149

[23] Ke Y Rao J Zhao H Lu Y Xiao N Yang Y. Accurate prediction of genome-wide RNA secondary structure profile based on extreme gradient boosting. Bioinformatics. (2019) 36:4576–82. 10.1093/bioinformatics/btaa534 32467966

[24] Lv X Chen J Lu Y Chen Z Xiao N Yang Y. Accurately predicting mutation-caused stability changes from protein sequences using extreme gradient boosting. J Chem Inf Model. (2020) 60:2388–95. 10.1021/acs.jcim.0c00064 32203653

[25] Al'Aref SJ Maliakal G Singh G van Rosendael AR Ma X Xu Z. Machine learning of clinical variables and coronary artery calcium scoring for the prediction of obstructive coronary artery disease on coronary computed tomography angiography: analysis from the CONFIRM registry. Eur Heart J. (2020) 41:359–67. 10.1093/eurheartj/ehz565 31513271 PMC7849944

[26] Tse G Lee S Zhou J Liu T Wong ICK Mak C. Territory-wide Chinese cohort of long QT syndrome: random survival forest and Cox analyses. Front Cardiovasc Med. (2021) 8:608592. 10.3389/fcvm.2021.608592 33614747 PMC7892622

[27] Chinese Society of Cardiology of Chinese Medical Chinese Myocarditis and Cardiomyopathy Association. Chinese guidelines for the diagnosis and treatment of dilated cardiomyopathy. J Clin Cardiol. (2018) 34:421–34. 10.13201/j.issn.1001-1439.2018.05.001

[28] Heart Failure Group of Chinese Society of Cardiology of Chinese Medical Association Chinese Heart Failure Association of Chinese Medical Doctor Association Editorial Board of Chinese Journal of Cardiology. Chinese guidelines for the diagnosis and treatment of heart failure 2018. Zhonghua Xin Xue Guan Bing Za Zhi. (2018) 46:760–89. 10.3760/cma.j.issn.0253-3758.2018.10.004 30369168

[29] Schiller NB Shah PM Crawford M DeMaria A Devereux R Feigenbaum H. Recommendations for quantitation of the left ventricle by two-dimensional echocardiography. J Am Soc Echocardiogr. (1989) 2:358–67. 10.1016/S0894-7317(89)80014-8 2698218

[30] Marwick TH Gillebert TC Aurigemma G Chirinos J Derumeaux G Galderisi M. Recommendations on the use of echocardiography in adult hypertension: a report from the European association of cardiovascular imaging (EACVI) and the American society of echocardiography (ASE)†. Eur Heart J Cardiovasc Imaging. (2015) 16:577–605. 10.1093/ehjci/jev076 25995329

[31] Verbraecken J Van de Heyning P De Backer W Van Gaal L. Body surface area in normal-weight, overweight, and obese adults. A comparison study. Metabolism. (2006) 55:515–24. 10.1016/j.metabol.2005.11.004 16546483

[32] Levey AS. A simplified equation to predict glomerular filtration rate from serum creatinine. J Am Soc Nephrol. (2000) 11:A0828. Available online at: https://hero.epa.gov/hero/index.cfm/reference/details/reference_id/658418

[33] Breiman L. Random forests. Mach Learn. (2001) 45:5–32. 10.1023/A:1010933404324

[34] Lee S Lee H Abbeel P Ng A. Efficient L~ 1 regularized logistic regression. In: Proceedings, the Twenty-First National Conference on Artificial Intelligence and the Eighteenth Innovative Applications of Artificial Intelligence Conference. Boston, MA: AAAI Press (2006). p. 401–8.

[35] Zadrozny B Elkan C. Transforming Classifier Scores into Accurate Multiclass Probability Estimates. Edmonton, AB: KDD (2002). p. 694–9. 10.1145/775047.775151

[36] Moliner-Abós C Mojón Álvarez D Rivas-Lasarte M Belarte LC Pamies Besora J Solé-González E. A simple score to identify super-responders to sacubitril/valsartan in ambulatory patients with heart failure. Front Physiol. (2021) 12:642117. 10.3389/fphys.2021.642117 33679455 PMC7930570

[37] Bielecka-Dabrowa A von Haehling S Aronow WS Ahmed MI Rysz J Banach M. Heart failure biomarkers in patients with dilated cardiomyopathy. Int J Cardiol. (2013) 168:2404–10. 10.1016/j.ijcard.2013.01.157 23416015

[38] Chatterjee NA Singh JP Szymonifka J Deaño RC Thai W Wai B. Incremental value of cystatin C over conventional renal metrics for predicting clinical response and outcomes in cardiac resynchronization therapy: the BIOCRT study. Int J Cardiol. (2016) 205:43–9. 10.1016/j.ijcard.2015.12.002 26710332 PMC4718799

[39] Xie L Terrand J Xu B Tsaprailis G Boyer J Chen QM. Cystatin C increases in cardiac injury: a role in extracellular matrix protein modulation. Cardiovasc Res. (2010) 87:628–35. 10.1093/cvr/cvq138 20489058 PMC2920813

[40] Huerta A López B Ravassa S José GS Querejeta R Beloqui Ó. Association of cystatin C with heart failure with preserved ejection fraction in elderly hypertensive patients: potential role of altered collagen metabolism. J Hypertens. (2016) 34:130–8. 10.1097/HJH.0000000000000757 26575701

[41] Barison A Aimo A Ortalda A Todiere G Grigoratos C Passino C. Late gadolinium enhancement as a predictor of functional recovery, need for defibrillator implantation and prognosis in non-ischemic dilated cardiomyopathy. Int J Cardiol. (2018) 250:195–200. 10.1016/j.ijcard.2017.10.043 29107357

[42] La Vecchia L Varotto L Zanolla L Spadaro GL Fontanelli A. Right ventricular function predicts transplant-free survival in idiopathic dilated cardiomyopathy. J Cardiovasc Med. (2006) 7:706–10. 10.2459/01.JCM.0000243006.90170.ce 16932086

[43] Merlo M Gobbo M Stolfo D Losurdo P Ramani F Barbati G. The prognostic impact of the evolution of RV function in idiopathic DCM. JACC Cardiovasc Imaging. (2016) 9:1034–42. 10.1016/j.jcmg.2016.01.027 27344413

[44] La Vecchia L Zanolla L Varotto L Bonanno C Spadaro GL Ometto R. Reduced right ventricular ejection fraction as a marker for idiopathic dilated cardiomyopathy compared with ischemic left ventricular dysfunction. Am Heart J. (2001) 142:181–9. 10.1067/mhj.2001.116071 11431676

[45] Emmens JE Jones DJL Cao TH Chan DCS Romaine SPR Quinn PA. Proteomic diversity of high-density lipoprotein explains its association with clinical outcome in patients with heart failure. Eur J Heart Fail. (2017) 20:260–7. 10.1002/ejhf.1101 29251807

[46] Freitas HFG Barbosa EA Rosa FHFP Lima ACP Mansur AJ. Association of HDL cholesterol and triglycerides with mortality in patients with heart failure. Braz J Med Biol Res. (2009) 42:420–5. 10.1590/S0100-879X2009000500004 19377790

[47] Toth PP Barter PJ Rosenson RS Boden WE Chapman MJ Cuchel M. High-density lipoproteins: a consensus statement from the national lipid association. J Clin Lipidol. (2013) 7:484–525. 10.1016/j.jacl.2013.08.001 24079290

[48] Kingwell BA Chapman MJ Kontush A Miller NE. HDL-targeted therapies: progress, failures and future. Nat Rev Drug Discov. (2014) 13:445–64. 10.1038/nrd4279 24854407

[49] McGarrah RW. Refocusing the AIM on HDL in the metabolic syndrome. Atherosclerosis. (2016) 251:531–3. 10.1016/j.atherosclerosis.2016.06.051 27397735 PMC5504691

[50] Sampietro T Neglia D Bionda A Dal Pino B Bigazzi F Puntoni M. Inflammatory markers and serum lipids in idiopathic dilated cardiomyopathy. Am J Cardiol. (2005) 96:1718–20. 10.1016/j.amjcard.2005.07.093 16360363

[51] Weber M Hamm C. Role of B-type natriuretic peptide (BNP) and NT-proBNP in clinical routine. Heart. (2006) 92:843–9. 10.1136/hrt.2005.071233 16698841 PMC1860679

