# Supplementary material for: Early Prediction of Left Ventricular Reverse Remodeling in First-Diagnosed Idiopathic Dilated Cardiomyopathy: A Comparison of Linear Model, Random Forest, and Extreme Gradient Boosting
Source: Front Cardiovasc Med. 2021 Aug 4;8:684004. doi: 10.3389/fcvm.2021.684004 (PMC8371915; doi:10.3389/fcvm.2021.684004)
Supplement: Supplementary file 1 [file Table_1.DOCX]

Supplementary Material

**Supplemental Figure 1.** Distribution of features in LVRR and non-LVRR. (**A**) Distribution density curves and proportional graphs of features with demographic variables in LVRR and non-LVRR; (**B**) Distribution density curves of features in laboratory variables with LVRR and non-LVRR; (**C**) Distribution density curves of features in electrocardiogram data with LVRR and non-LVRR; (**D**) Distribution density curves and proportional graphs of features in echocardiographic data with LVRR and non-LVRR. The feature names in red are those features showing a statistically significant difference in LVRR and non-LVRR. The dotted lines that are parallel to the y-axis are the mean of each numerical feature in two groups

ACEIs = angiotensin-converting enzyme inhibitors; ADA = adenosine deaminase; AF = atrial fibrillation; ALB = albumin; ALT = alanine aminotransferase; AOR = aortic root diameter; APTT = activated partial thromboplastin time; ARBs = angiotensin receptor blockers; ARNIs = angiotensin receptor-neprilysin inhibitors; AST = aspartate aminotransferase; AVB = atrioventricular block; BMI = body mass index; CA = serum free calcium; Cl = serum chloride; CKMB = creatine kinase MB; CO2 = carbon dioxide combining power; CYSC = cystatin C; DBP = diastolic blood pressure; EF = ejection fraction; eGFR = estimated glomerular filtration rate; FT3 = free T3; FT4 = free T4; GGT = gamma-glutamyl transpeptidase; FB = fibrinogen; FBG = fasting blood glucose; FFA = non-esterified fatty acid; HbA1c = hemoglobin A1c; HDL-C = high-density lipoprotein cholesterol; HGB = hemoglobin; HR = heart rate; ICD = implantable cardioverter defibrillator; INR = international normalized ratio; IVSD = interventricular septal dimension; K = serum potassium; LA = left atrial; LDL-C = low-density lipoprotein cholesterol; LVDD = left ventricular end-diastolic dimension; LVMI = left ventricular mass index; LVPWD = LVPWd = left ventricular posterior wall dimension; LVRR = left ventricular reverse remodeling; LYM_COUNT = lymphocyte count; MONO-COUNT = monocyte count; MR = mitral regurgitation; MRA = mineralocorticoid receptor antagonist; Na = serum sodium; NEU_COUNT = neutrophil count; NUM_APB = number of atrial premature beat in 24h; NUM_VPB = number of ventricular premature beat in 24h; NYHA = New York Heart Association; P = serum phosphorus; PLT = platelet; PR_DUR = PR duration; PT = prothrombin time; PTA = prothrombin time activity; QRS_DUR = QRS duration; QTC_DUR = QTc duration; RDW = red cell distribution width; RVDD = right ventricular end-diastolic dimension; RWT = relative wall thickness; SBP = systolic blood pressure; SOD = superoxide dismutase; TG = triglyceride; TR = tricuspid regurgitation; TSH = thyroid stimulating hormone; UA = uric acid; VF = ventricular fibrillation; VT = ventricular tachycardia; WBC = white blood cell


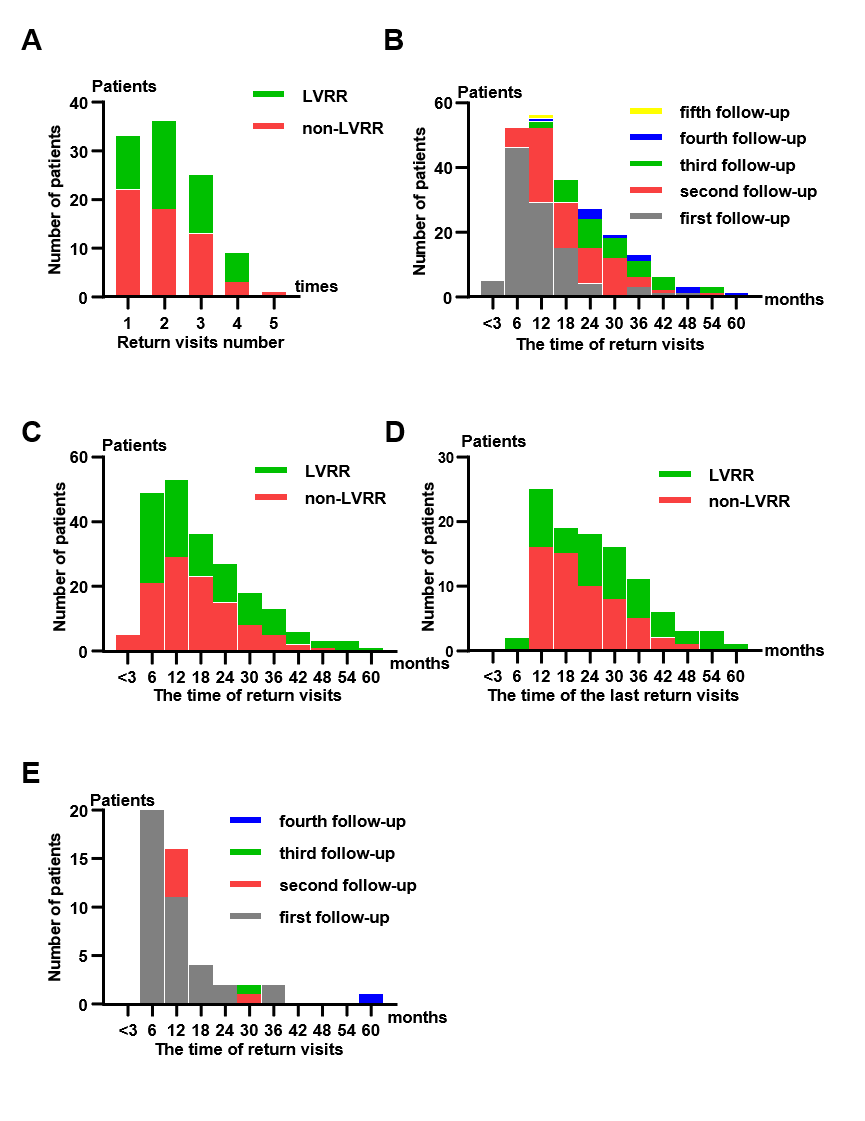


**Supplemental Figure 2.** Distribution characteristics of data from return visits. (**A**) The distribution of the return visit numbers in the two groups. (**B**) The time distribution of all data from return visits. (**C**) The number of patients who had information from return visits in different time intervals. (**D**) The time distribution of the last return visit in the two groups. (**E**) The time distribution of the first time the LVRR standard was met in the LVRR group

LVRR = left ventricular reverse remodeling





**Supplemental Figure 3.** Receiver operating characteristic curve of XGBoost model in training and testing sets. Solid lines were validated by the testing set, while dashed lines were validated by the training set


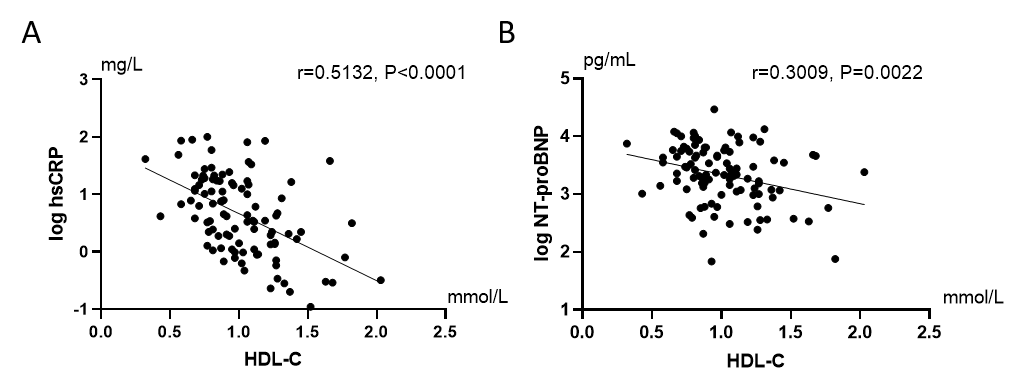


**Supplemental Figure 4.** Correlations among NT-proBNP, HDL-C and inflammatory indicators. (**A**) Relationship between the Lg hsCRP and HDL-C levels. The Lg hsCRP and HDL-C levels are represented as scatter plots. The black line indicates the linear regression curve (both near and below). (**B**) Relationship between the Lg NT-proBNP and HDL-C level

**Supplemental Table 1.** List of Candidate Variables Collected to Predict Left Ventricular Reverse Remodeling in Patients with Dilated Cardiomyopathy

| **Variable Names** | **Definition** |
| --- | --- |
| age_entry | Age first diagnosed idiopathic dilated cardiomyopathy |
| GENDER | Gender of the subject |
| BMI | Body mass index |
| FOLLOW_TIMES | Total follow-up times |
| LAST_MONTHS | Last follow-up time |
| SMOKE_EVER | Has subject ever been a smoker |
| SMOKE_YEARS | How many years since smoking |
| alcohol_cat | Has subject ever been a drinker |
| alcohol_years | How many years since drinking |
| HTN | Hypertension |
| HTN_LEVEL | Stage 1 vs 2 vs 3 of hypertension |
| HTN_YEARS | Duration of hypertension |
| DM | Diabetes Mellitus |
| DM_YEARS | Duration of diabetes |
| AF | Atrial fibrillation |
| AF_TYPE | Atrial fibrillation: paroxysmal vs persistent |
| VTVF | Ventricular tachycardia or ventricular fibrillation |
| VERSION | Drug vs Electrical |
| STROKE | Previous Stroke |
| ICD | Implanted cardioverter defibrillator |
| nyha_class | NYHA class 1 vs 2 vs 3 vs 4 |
| HR | Heart rate |
| SBP | Systolic blood pressure |
| DBP | Diastolic blood pressure |
| EF | Left ventricular ejection fraction |
| LVDD | Left ventricular end-diastolic dimension |
| LVPWD | Left ventricular posterior wall dimension |
| AOR | Aortic root dimension |
| LA | Left atrial dimension |
| RVDD | Right ventricular end-diastolic dimension |
| IVSD | Interventricular septal dimension |
| RWT | Relative wall thickness |
| LVMI | Left ventricular mass index (g/m^2^) |
| MR_SEVERE | Severe mitral regurgitation |
| TR | Moderate and severe tricuspid regurgitation |
| QRS_DUR | QRS duration |
| PR_DUR | PR duration |
| QTC_DUR | QTc duration |
| ECG_LBBB | Left bundle branch block - Yes/No indicator |
| ECG_AVB | Atrial ventricular block – Yes/No |
| NUM_VPB | Number of ventricular premature beat in 24 h |
| NUM_APB | Number of atrial premature beat in 24 h |
| WBC | White blood cell count |
| HGB | Hemoglobin |
| PLT | Platelet count |
| RDW | Red cell distribution width |
| LYM_COUNT | Lymphocyte count |
| NEU_COUNT | Neutrophil count |
| MONO_COUNT | Monocyte count |
| PT | Prothrombin time |
| PTA | Prothrombin time activity |
| INR | International normalized ratio |
| FB | Fibrinogen |
| APTT | Activated partial thromboplastin time |
| D_dimer | D-dimer level |
| NT_PROBNP | N-terminal pro brain natriuretic peptide |
| TNTHS | High-sensitivity troponin T |
| ALT | Alanine aminotransferase |
| AST | Aspartate transaminase |
| GGT | Gamma-glutamyl transpeptidase |
| K | Serum potassium |
| Na | Serum sodium |
| CI | Serum chloride |
| CA | Serum calcium adjusted by albumin |
| P | Serum phosphorus |
| BUN | Blood urea nitrogen |
| CO_2_ | Carbon dioxide combining power |
| eGFR | Estimate glomerular filtration rate |
| CysC | Cystatin C |
| UA | Uric Acid |
| FBG | Fasting blood glucose |
| CHOL | Total cholesterol |
| TG | Triglyceride |
| HDL-C | High-density lipoprotein cholesterol |
| LDL-C | Low density lipoprotein cholesterol |
| ALB | Albumin |
| CKMB | Creatine kinase MB |
| hsCRP | High-density C-reactive protein |
| FFA | Non-esterified fatty acid |
| ADA | Adenosine deaminase |
| SOD | Superoxide dismutase |
| HbA1c | Hemoglobin A1c |
| FT3 | Free T3 |
| FT4 | Free T4 |
| TSH | Thyroid stimulating hormone |
| ACEI_ARB_ARNI | Treatment of ACEI or ARB or ARNI |
| DOS_ACEI | Percentage of target dosage of ACEI or ARB or ARNI |
| ADD_ACEI | Add dosage of ACEI or ARB or ARNI: Yes or No |
| Beta_blocker | Treatment of Beta blocker |
| DOS_Beta | Percentage of target dosage of Beta blocker |
| ADD_Beta | Add dosage of Beta blocker: Yes or No |
| MRA | Treatment of spirolactone |
| DIU | Treatment of diuretic |
| DIGOXIN | Treatment of digoxin |
| STATIN | Treatment of statin |
| ANTI_COA | Treatment of anticoagulation |
| ANTI_PLT | Treatment of antiplatelet |
| AMIO | Treatment of amiodarone |
| TRIME | Treatment of trimetazidine |
| IVAB | Treatment of ivabradine |
